# A Protective Role for Arachidonic Acid Metabolites against Advanced Colorectal Adenoma in a Phase III Trial of Selenium

**DOI:** 10.3390/nu13113877

**Published:** 2021-10-29

**Authors:** Jessica A. Martinez, Meghan B. Skiba, H-H. Sherry Chow, Wade M. Chew, Kathylynn Saboda, Peter Lance, Nathan A. Ellis, Elizabeth T. Jacobs

**Affiliations:** 1 Department of Nutritional Sciences, University of Arizona, Tucson, AZ 85721, USA; 2 University of Arizona Cancer Center, Tucson, AZ 85724, USA; schow@arizona.edu (H.-H.S.C.); wchew@arizona.edu (W.M.C.); ksaboda@arizona.edu (K.S.); mplance@arizona.edu (P.L.); naellis@arizona.edu (N.A.E.); jacobse@arizona.edu (E.T.J.); 3 Knight Cancer Institute, Oregon Health & Science University, Portland, OR 97201, USA; ski-ba@ohsu.edu; 4 Department of Molecular and Cellular Biology, University of Arizona, Tucson, AZ 85724, USA

**Keywords:** colorectal adenoma, oxylipins, selenium, colorectal neoplasia, colon cancer, ARA, arachidonic acid

## Abstract

Oxylipins derived from arachidonic acid (ARA) have been implicated in the development of colorectal adenomas and colorectal cancer. The primary purpose of this work was to determine the relationship between plasma levels of oxylipins and colorectal adenoma characteristics at study entry, as well as with the development of a new adenoma during follow-up within a Phase III adenoma prevention clinical trial with selenium (Sel). Secondarily, we sought to determine whether the selenium intervention influenced plasma oxylipin levels. Four oxylipins were quantified in stored plasma samples from a subset of Sel study subjects (*n* = 256) at baseline and at 12-months. There were significantly lower odds of an advanced adenoma at baseline with higher prostaglandin E_2_ (PGE_2_), with an OR (95% CI) of 0.55 (0.33–0.92), and with 5-hydroxyeicosatetraenoic acid (5-HETE) ((0.53 (0.33–0.94)); and of a large adenoma with higher PGE_2_ ((0.52 (0.31–0.87)). In contrast, no associations were observed between any oxylipin and the development of a new adenoma during follow-up. Selenium supplementation was associated with a significantly smaller increase in 5-HETE after 12 months compared to the placebo, though no other results were statistically significant. The ARA-derived oxylipins may have a role in the progression of non-advanced adenoma to advanced, but not with the development of a new adenoma.

## 1. Introduction

Oxylipins are the oxygenated lipid metabolites of ω-3 and ω-6 fatty acids. Enzymatic production of oxylipins is achieved via the cyclooxygenase(COX), lipoxygenase (LOX), and cytochrome P450 (CYP450) enzymes. While oxylipins have a broad range of potent biological activities, many of the oxylipins produced from the ω-6 fatty acid, arachidonic acid (ARA) are pro-inflammatory and pro-tumorigenic with prostaglandin E_2_ (PGE_2_) being the most thoroughly studied in relation to colorectal and other cancers [1]. Dietary consumption of a Western style eating pattern containing high a ω-6 to ω-3 ratio has been associated with higher risk of colorectal cancer (CRC) [2] and colorectal adenomas (CRA) [3]. Clinical trials with nonsteroidal anti-inflammatory drugs (NSAIDs) and aspirin, inhibitors of cyclooxygenase-2 (COX-2), have demonstrated success in the prevention of colorectal adenomas via reduction in PGE_2_ [4]. Further supporting the role of PGE_2_ in colorectal carcinogenesis is that the prostaglandin E_2_ metabolite (PGEM) was observed to be associated with increased risk for advanced adenoma among women, but not non-advanced, in a nested case-control of the Tennessee Colorectal Polyp Study; there was no reduction in risk for men [5]. The PGEM was also related to significantly greater odds for high-risk adenoma in a nested case control in the Nurses’ Health Study, and regular use of NSAIDs was associated with significant reduction in risk among women with high baseline PGEM [6]. In a nested case control study conducted within the Shanghai Women’s Health Study, the relative risks of developing CRC were significantly elevated with increasing quartiles of urinary PGEM levels [7]. 

The overall role for the COX-2/PGE_2_ pathway in colorectal carcinogenesis is well characterized [8]; however possible differential effects of the pathway in the development of non-advanced versus advanced CRAs (i.e., adenomas ≥1 cm, villous histology, or high-grade dysplasia) have not been well defined. Individuals who develop an advanced CRA, or multiple non-advanced CRA, are more likely to develop CRC [9]. Thus, screening guidelines recommend more frequent surveillance colonoscopies after diagnosis and removal of advanced CRAs or multiple CRAs than for non-advanced CRAs [10]. More specific screening based on molecular signatures would allow for more targeted prevention and to optimize the timing of surveillance colonoscopy. 

**Figure 1:**
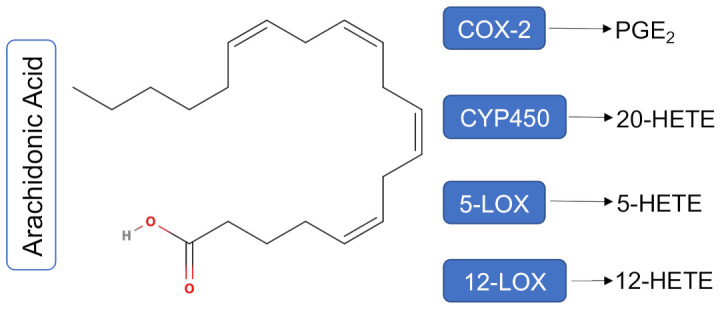
Arachidonic Acid metabolism via COX-2, CYP450, 5-LOX and 12-LOX. ARA metabolism via COX-2, CYP450, 5-LOX and 12-LOX produces PGE_2_, 20-HETE, 5-HETE, and 12-HETE respectively. Only those oxylipins quantified on our platform are illustrated here. **Abbreviations**: COX-2: cyclooxygenase-2; CYP450: cytochrome P450; 5-LOX: 5-lipoxygenase; 12-LOX: 12-lipoxygenase; PGE_2_: prostaglandin E_2_; 5-HETE: 5-hydroxyeicosatetraenoic acid; 12-HETE: 12-hydroxyeicosatetraenoic acid.

Genetic polymorphisms in the COX-2/PGE_2_ pathway appear to contribute to the development of colorectal adenomas and influence the interval time to adenomas recurrence [11]; however, the biological mechanisms determining the progression from an adenomatous polyp to colorectal cancer are still poorly understood. Genetic variations in the LOX pathway have also been shown to influence CRC and CRA risk [12], and cytochrome P450 4A/4F CYP4A/4F [the CYP450 enzyme that produces 20-hydroxyeicosatetraenoic acid (20-HETE)] is elevated in multiple cancers, including CRC [13]. Given that each of these enzymes produces multiple oxylipins, it is likely that the entire ARA cascade is involved in the progression from CRA to CRC. Figure 1 illustrates the ARA metabolism via COX-2, CYP4A/4F, 5-lipoxygenase (5-LOX) and 12-lipoxygenase (12-LOX), and to produce PGE_2_, 20-HETE, 5-HETE, and 12-HETE, respectively. Only those select oxylipins quantified on our platform are illustrated here. 

In the context of a Phase III colorectal adenoma prevention clinical trial with selenium, the main purpose of this work was to determine whether select oxylipins derived from ARA [PGE_2_, 5-HETE, 12-HETE, and 20-HETE] were related to the presence of advanced adenomas and features of advanced adenomas at baseline, as well as with the development of metachronous (new) adenoma. Secondarily, we sought to determine whether the selenium intervention, which resulted in a statistically significant reduction in recurrence of adenomas in individuals that entered the trial with an advanced adenoma [14], influenced oxylipin levels in the plasma. 

## 2. Materials and Methods

### 2.1. Study Population

The details of the Selenium Trial (Sel) have been previously described [14,15,16]. Briefly, this study was originally designed as a phase III, randomized, placebo controlled, two-by-two factorial trial of celecoxib (400 mg q.d.) crossed with selenium (200 μg q.d. as selenized yeast) for preventing colorectal adenomas (Clinical Trials.gov No. NCT00078897). Due to the reported coxib-associated cardiovascular toxicity at that time, the External Data and Safety Monitoring Committee (EDSMC), recommended suspension of the celecoxib arm [17,18]. The trial was modified to a two-arm design comparing selenium with the placebo. Participants randomly assigned during the factorial phase were retained in the appropriate selenium or placebo arm, but were no longer allocated celecoxib or its placebo [14]. Here, stored plasma samples from a subset of study subjects (*n* = 256) that participated in the selenium and placebo arms of the Sel Trial were utilized to quantify concentrations of four oxylipins of ARA, selected for their potential effect in colorectal carcinogenesis, at baseline and 12-months. There were no participants selected from the celecoxib arm for this study. *A priori*, we calculated that to reach 80% statistical power to detect standardized differences of 0.29 and 0.45 for adenoma recurrence and advanced adenoma recurrence, respectively, we would require 125 individuals with non-advanced baseline lesions and 125 with advanced lesions, provided that each group had half of the participants in the placebo group and half in the selenium group. Thus, participants with available data included 126 individuals who had an advanced lesion and 130 who had a non-advanced adenoma at baseline. Of the individuals with an advanced lesion, 62 (49.2%) were from the placebo group and 64 (50.8) were in the selenium group. Among those with a non-advanced lesion at baseline, 69 (53.1%) and 61 (46.9%) were randomized to the placebo and selenium groups, respectively.

### 2.2. Plasma Sample Collection and Preparation

Previously collected plasma samples had been immediately stored at −80 °C and were not thawed prior to this work. Plasma samples were prepared as previously described [19]. Briefly, once thawed, triphenylphosphine and butylated hydroxytoluene (0.2% *w*/*w*) were added to 250 μL of plasma to stabilize the oxylipins. The sample was then spiked with a set of deuterated isomers of four target analytes (PGE_2_-d_4_, 5-HETE-d_8_, 12-HETE-d_8_, and 20-HETE-d_6_) contained in 10 μL of methanol and was then subjected to solid phase extraction. The collected eluents were evaporated to dryness using a centrifugal vacuum concentrator and re-constituted with 50 μL of methanol solution with 1-cyclohexyl-dodecanoic acid urea as an internal standard. The spiked samples were vortexed and centrifuged before transfer to high performance liquid chromatography (HPLC) vials for analysis.

### 2.3. Reverse Phase Chromatography with HPLC-MS

The PGE_2_, 5-HETE, 12-HETE, and 20-HETE quantification was performed on an Agilent Ultivo QQQ MS system coupled to an Agilent 1290 Infinity II UPLC system (Agilent, Santa Clara, CA, USA). Chromatographic separation of oxylipins was achieved using a gradient of water, methanol, and acetonitrile all with 0.1% acetic acid (*v*/*v*). Acquisition parameters were as previously described with minor modifications [20]. The acquired data were quantified by Quant-My-Way (Agilent, Santa Clara, CA, USA) using calibration curves.

### 2.4. Statistical Analysis

Data underwent a quality check to manually inspect for detected values compared to the lowest standard detected for each date of analysis and for individual oxylipins; values of true zero were confirmed. For peaks below the limit of quantification (LOQ), values were transformed using LOQ/2, by date, consistent with previous studies [21,22]. Unadjusted generalized linear mixed models were performed using log transformed oxylipin concentrations to test for batch effect by date. No batch effect was found. Concentrations were graphed using two-way parallel coordinate plots by adenoma status to evaluate outliers; the outlying values were consistent and not related to batch date. 

Baseline descriptive characteristics by adenoma status were completed using Two-sample t-tests, Pearson’s chi-squared tests, or Fisher’s exact tests as indicated. We employed Student’s t-tests to investigate between group differences for change in oxylipin concentration by randomization arm and within the selenium arm and placebo arms. Using cut points set at the median value for each metabolite concentration, adjusted multinomial logistic regression models were conducted; the latter were adjusted for face validity variables including age, sex, and self-reported NSAID use. To investigate baseline oxylipin concentration and associations with baseline adenoma histology (advanced, ≥1 cm, villous, and multiplicity) and change in oxylipin concentration association with follow-up adenoma status (non-advanced or advanced) by randomization arm and overall, we employed logistic regression models. Individuals with available endpoint data for newly found adenomas were included. Peaks below LOQ were transformed using LOQ/2. Values are presented as pg/mL for interpretation. All analyses were completed in STATA 16.1 (StataCorp, LLC., College Station, TX, USA), with an alpha level of 5% for statistical significance. 

## 3. Results

Table 1 presents the baseline characteristics of the study participants by baseline adenoma status (advanced vs. non-advanced). Participants with an advanced or non-advanced adenoma at baseline were similar in terms of sex, age, education level, body mass index (BMI), smoking status, NSAID, and aspirin use, as well as relatives with CRC and history of polyps or cancer (Table 1). The number of participants randomized to the selenium intervention was also balanced between groups. There was no difference at baseline between groups in ω-6 or ω-3 fatty acid intake, but individuals in the non-advanced adenoma group entered the original clinical trial with higher blood selenium concentration at baseline than the advanced group (140.0 (±26.2) versus 132.5 (±22.3)).

Table 2 presents the odds ratios (95% CI) for characteristics of baseline adenoma by high (at or above median) vs. low (below median) concentrations of oxylipins at baseline using a cross-sectional study design. These results show that those above the median for PGE_2_ were statistically significantly less likely to have an advanced adenoma at baseline, with an OR (95% CI) of 0.55 (0.33–0.92) compared to those below the median. Similarly, those with baseline concentrations of 5-HETE that were above the median also had statistically significantly lower odds of having an advanced adenoma (OR = 0.53; 95% CI 0.33–0.94). We next considered the relationship between oxylipins and specific features of advanced adenoma (size ≥1 cm; ≥25% villous histology, or presence of 3 or more non-advanced adenomas). The PGE_2_ was significantly inversely associated with the presence of a large adenoma (OR = 0.52; 95% CI: 0.31–0.87). Finally, those with higher concentrations of 5-HETE at baseline also had significantly lower odds of developing an adenoma with villous histology (OR = 0.37; 95% CI: 0.19–0.75). These results are in contrast to those shown in Table 3, where there was no significant relationship between oxylipins and any adenoma outcomes. When evaluating advanced and non-advanced adenoma groups together, there was no association between change in any oxylipins and the new adenoma outcome (data not shown). 

There were significant increases in PGE_2_ (0.39 ± 1.38l pg/mL *p* < 0.001), 12-HETE 2.48 ± 12.13 pg/mL *p* = 0.001), and 5-HETE (60.32 ± 282.31 pg/mL; *p* < 0.001) over the study duration (Table 4), but no change in 20-HETE. It has been suggested that selenium can suppress COX-2 via off-target effects [23]. 

Therefore, we next sought to determine whether changes in oxylipins differed by treatment group (Table 5). No statistically significant differences were detected for PGE_2_, 20-HETE, or 12-HETE. However, for 5-HETE, those in the placebo group exhibited a significantly greater mean increase over time of 99.1 ± 381.9 pg/mL, compared to those in the selenium group (19.3 ± 84.1 pg/mL; *p* = 0.02). 

Given that >50% of the cohort was regularly taking NSAIDs, which directly suppress COX-2 and thus could mask any effect of selenium on PGE_2_, we conducted a sensitivity analysis among the non-NSAID users. However, there were no significant differences in the magnitude of change for any oxylipins with selenium compared to the placebo (data not shown). When comparing NSAID users vs. non-users in the placebo group only, we observed no differences in the magnitude of change for any of the oxylipins (data not shown).

## 4. Discussion

Given that PGE_2_ has demonstrated a role in the initiation, promotion, and progression phases of colorectal carcinogenesis [8], we hypothesized that higher circulating concentrations of PGE_2_ and other pro-inflammatory products of ARA would be related to the presence of advanced adenoma features compared to those with lower concentrations. However, in the present study, we observed the opposite; that higher concentrations of PGE_2_ and the 5-LOX metabolite 5-HETE were significantly inversely associated with the presence of advanced adenomas at baseline. However, no statistically significant relationships were observed for any oxylipin and the odds of developing a new adenoma. 

Our results suggest that the utility of oxylipins as a biomarker of risk for future adenoma may depend on baseline adenoma status. Case-control studies have supported PGEM, the urinary marker of PGE_2_, as a biomarker of risk for advanced CRA to non-advanced adenomas [24], or polyp-free controls [5,25]. Interestingly, however, Ghandimi et al. showed decreased odds for any CRA among women, but no effect in men, with higher serum ARA levels in a case-control study [26] which is in line with our results of an inverse association between ARA products and advanced adenoma features at baseline. Similarly, Hall et al. showed non-significant decreased risk for CRC with increased total whole blood levels of ω-6, and a significantly reduced risk with increased ω-3 levels [27]. Kojima et al. showed no increased risk of CRC with higher quartiles of serum ARA in the Japan Collaborative Cohort Study, but did not evaluate CRA [28]. Conversely, Pot et al. showed a positive association between ARA or ω-6 and increased CRA risk in a case-control study [29]. Rifkin et al. quantified ARA in red blood cells and showed a strong association between ARA and advanced adenoma risk compared to polyp-free controls [30]. Taken together, these studies indicate a potential duality of function for ARA and its metabolites in adenoma risk such that overall healthy individuals might receive protection from higher ARA, but once other adenoma-promoting factors are introduced an increase in ARA may promote the tumorigenic environment. 

Oxylipins derived from the LOX and P450 arms of the ARA pathway have been less studied in the context of CRA. Concentrations of the 5-LOX metabolite, 5-HETE, have been shown to be higher among patients with adenomas as compared to those without [31]. In the current study higher 5-HETE levels were significantly associated with lower odds for advanced and villous adenomas at baseline, but not at follow-up. Whether this association with baseline adenomas is a mechanistic role or simply reflective of higher ARA in this population of individuals who had already developed at least one colorectal adenoma is unknown. Overall, our study suggests a protective role of ARA products against the formation of a primary advanced adenoma, but not for the development of a new adenoma. 

It has been suggested that selenium has an inhibitory effect on COX-2, and thus oxylipins, through off-target mechanisms. Hwang et al. found that AMP-activated protein kinase (AMPK) mediated the anticancer effects of selenium via a COX-2/prostaglandin E_2_ signaling pathway [32]. Se-treated cells have decreased COX-2 and nuclear factor (NF)-ΚB activity with associated changes in prostaglandins [32,33,34,35,36], and Se deficiency in animals is associated with upregulation of inducible nitric oxide synthesis and COX-2 [34]. Given that NSAIDs and aspirin are potent COX-2 inhibitors, and ~50% of the study cohort was already taking and continued to take aspirin, we expected that selenium supplementation would not have further suppression effects. As expected, there was no decrease in PGE_2_ with selenium supplementation in the overall cohort; however, there were also no significant differences when participants not regularly taking NSAIDs were analyzed separately.

Selenoproteins have been shown to inhibit both 5-LOX and 12-LOX enzymes in different cell types [37]. In prostate cancer cell lines, Se induced apoptosis through decreases in 5-LOX metabolites [38]. In line with this evidence there was a significantly greater increase in 5-HETE in the placebo compared to the selenium group.

This study had several strengths including that samples were taken from a large, placebo-controlled colorectal adenoma prevention trial with selenium. Another strength of this study is that participants had to have an adenoma at study entry; therefore, there was a large percentage of participants with a recurrent adenoma. Our results showing a decreased risk of advanced adenoma at baseline with higher pro-inflammatory oxylipins is opposite of what we hypothesized; therefore, we considered the possibility that our results were due to a systematic technical error. We conducted an extensive day to day quality check of the data and there was no systematic pattern detected. Samples from participants with advanced and non-advanced adenomas were randomized throughout the runs as well as pre- and post-intervention samples. Individual outliers were thoroughly examined for peak quality. Internal standard peaks were also checked. Standard curves and LOQs were inspected for each run day. Antioxidant was added when the samples were thawed to stabilize the oxylipins, and samples never went through multiple freeze-thaw cycles. Another limitation includes small sample sizes in subgroup analyses, which precluded further sub-analyses. Additionally, follow-up oxylipins were quantified at 12 months, which results in differential time periods between blood draw and the development of a new adenoma; this likely attenuated our ability to detect an association between the change in oxylipins and outcomes. Finally, for this project, we had only one measure of each oxylipin at baseline and one at follow-up. It is possible that a single measure at each timepoint does not fully capture an individual’s usual concentrations of these metabolites.

## 5. Conclusions

This study suggests a protective effect for the oxylipins PGE_2_ and 5-HETE against advanced adenoma at baseline, and this relationship seems to be driven by large adenomas. Importantly, selenium suppressed 5-HETE relative to the placebo from baseline to 12-months. However, we were unable to determine whether the protective effect of selenium for individuals that entered the Sel trial with an advanced adenoma [14] can be explained by any effect of selenium on oxylipins due to small sample sizes in the subgroup analysis. Plasma levels of these oxylipins were lower in our study compared to studies of colorectal cancer. Future studies should investigate the possibility of a duality of function for these oxylipins with both very low and very high levels increasing risk for advanced adenoma and cancer, but moderate levels providing the appropriate amount of inflammatory signaling to initiate a protective cascade.

## Figures and Tables

**Table 1:** Baseline demographic characteristics of individuals participating in the selenium rrial by baseline adenoma status (*n* = 256).

	Non-Advanced	Advanced	*p*-Value
	*n* = 130	*n* = 126	
Age (years) ^a^	63.3 (8.7)	61.6 (8.3)	0.12
Sex ^b^			0.15
Female	48 (36.9%)	36 (28.6%)	
Male	82 (63.1%)	90 (71.4%)	
Highest level of education ^b^			0.94
High School or less	25 (19.2%)	22 (17.5%)	
Some College	37 (28.5%)	40 (31.7%)	
Bachelor’s Degree	30 (23.1%)	29 (23.0%)	
Graduate or Professional Degree	38 (29.2%)	35 (27.8%)	
Baseline Body Mass Index (kg/m^2^) ^a^	28.8 (5.2)	28.7 (4.4)	0.78
Smoke cigarettes (≥100) ^b^			0.30
Current	8 (6.3%)	13 (10.7%)	
Never	58 (45.7%)	46 (38.0%)	
Past	61 (48.0%)	62 (51.2%)	
Routine NSAID use ^b^	6 (4.6%)	4 (3.2%)	0.55
Routine Aspirin use ^b^	60 (46.2%)	49 (38.9%)	0.24
CRC in a first-degree relative ^b^			0.18
None	96 (76.8%)	102 (83.6%)	
One or more	29 (23.2%)	20 (16.4%)	
History of polyps ^b^	49 (38.6%)	39 (31.2%)	0.22
History of cancer ^b,d^	8 (6.2%)	5 (4.0%)	0.43
Randomization ^c^			0.62
Placebo	69 (53.1%)	62 (49.2%)	
Selenium	61 (46.9%)	64 (50.8%)	
Serum Selenium Concentration (ng/mL) ^a^	140.0 (26.2)	132.5 (22.3)	0.014
Dietary Omega 6 (mg/day) ^a^	14.9 (9.1)	13.6 (9.9)	0.28
Dietary Omega 3 (mg/day) ^a^	1.5 (0.7)	1.3 (0.7)	0.16
Dietary Omega6/Omega3 Ratio) ^a^	9.9 (1.8)	9.9 (2.4)	0.97

Data are presented as mean (SD) or median (IQR) for continuous measures, and *n* (%) for categorical measures. ^a^ Two sample *t*-test; ^b^ Pearson’s chi-squared; ^c^ Fisher’s exact; ^d^ Excluding non-melanoma skin cancer; CRC: colorectal cancer; NSAIDs: Non-steroidal anti-inflammatory drugs.

**Table 2:** Odds of characteristics of baseline adenoma by high vs. low concentrations of baseline oxylipins (*n* = 256) ^a,b^.

Category of Oxylipin Metabolite	Advanced *n* = 126	Size ≥1 cm *n* = 115	Villous Histology *n* = 47	Multiplicity *n* = 50
PGE_2_ (pg/mL)	OR (95% CI)			
1 (0.00–18.0)	Ref	Ref	Ref	Ref
2 (18.1–277.5)	0.55 (0.33–0.92)	0.52 (0.31–0.87)	0.90 (0.47–1.72)	0.78 (0.41–1.48)
20-HETE (pg/mL)				
1 (0.0–5.0)	Ref	Ref	Ref	Ref
2 (5.1–20.3)	0.95 (0.57–1.57)	0.97 (0.58–1.61)	1.15 (0.60–2.19)	0.92 (0.49–1.74)
12-HETE (pg/mL)				
1 (0.0–24.5)	Ref	Ref	Ref	Ref
2 (24.6–718.7)	0.66 (0.40–1.09)	0. 84 (0.51–1.40)	0.71 (0.37–1.36)	0.80 (0.43–1.51)
5-HETE				
1 (0.0–46.6)	Ref	Ref	Ref	Ref
2 (46.7–301.2)	0.53 (0.33–0.94)	0.61 (0.37–1.02)	0.37 (0.19–0.75)	0.88 (0.47–1.67)

^a^ Categories created by dividing observations as < median vs. ≥ median value for each metabolite; ^b^ Logistic regression models adjusted for age, sex, and NSAID use. PGE_2_: prostaglandin E_2_; 20-HETE: 20-hydroxyeicosatetraenoic acid; 12-HETE: 12-hydroxyeicosatetraenoic acid; 5-HETE: 5-hydroxyeicosatetraenoic acid.

**Table 3:** Odds ratios (95% CIs) for the association between change in oxylipin concentrations and follow-up adenoma endpoints, overall and stratified by treatment group (*n* = 256).

Oxylipin	Overall ^a^ *n* = 256 (OR, 95% CI) ^d^		Selenium ^b^ *n* = 125 (OR, 95% CI)		Placebo ^c^ *n* = 131 (OR, 95% CI)	
Category of Metabolite Concentration	Non-Advanced	Advanced	Non-Advanced	Advanced	Non-Advanced	Advanced
PGE_2_ (pg/mL)						
1 (−2.8 to −0.0002)	Ref	Ref	Ref	Ref	Ref	Ref
2 (−0.0003 to 14.2)	1.41 (0.85–2.00)	1.46 (0.54–3.97)	1.54 (0.67–3.49)	0.64 (0.20–2.04)	1.24 (0.55–2.80)	0.72 (0.22–2.28)
20-HETE (pg/mL)						
1 (−0.004 to 0.00)	Ref	Ref	Ref	Ref	Ref	Ref
2 (0.01 to 11.9)	0.98 (0.54–1.77)	1.54 (0.69–3.45)	1.01 (0.43–2.36)	1.95 (0.61–6.25)	0.95 (0.40–2.19)	1.16 (0.36–2.49)
12-HETE (pg/mL)						
1 (−57.1 to 0.0)	Ref	Ref	Ref	Ref	Ref	Ref
2 (0.01 to 80.3)	1.40 (0.74–2.63)	1.30 (0.45–3.13)	1.09 (0.46–2.58)	0.83 (0.25–2.81)	1.55 (0.58–4.14)	2.29 (0.59–8.90)
5-HETE (pg/mL)						
1 (−300.8 to 0.00)	Ref	Ref	Ref	Ref	Ref	Ref
2 (0.01 to 2583.5)	1.21 (0.66–2.21)	1.33 (0.58–3.03)	0.97 (0.40–2.37)	1.67 (0.53–5.3)	1.62 (0.32–3.67)	1.09 (0.32–3.69)

^a^ Includes 126 individuals who had an advanced lesion and 130 who had a non-advanced adenoma at baseline. ^b^ Includes 64 individuals who had an advanced lesion and 61 participants who had a non-advanced adenoma at baseline. ^c^ Includes 62 individuals who had an advanced lesion and 69 participants who had a non-advanced adenoma at baseline. ^d^ Logistic regression models adjusted for age, sex, and NSAID use. Zero values transformed to LOQ/2. PGE_2_: prostaglandin E_2_; 20-HETE: 20-hydroxyeicosatetraenoic acid; 12-HETE: 12-hydroxyeicosatetraenoic acid; 5-HETE: 5-hydroxyeicosatetraenoic acid.

**Table 4:** Baseline and follow-up circulating oxylipin concentrations in sample overall (*n* = 256).

Mean ± SD	Baselinen *n* = 256	Follow-Up ^a^ *n* = 253	Differencen *n* = 253	*p*-Value ^b^
PGE_2_^c,d^	0.08 ± 0.32	0.48 ± 1.4	0.39 ± 1.38	<0.001
20-HETE	0.05 ± 0.04	0.05 ± 0.04	0.00 ± 0.03	0.68
12-HETE	1.4 ± 5.86	3.90 ± 12.0	2.48 ± 12.13	0.001
5-HETE	3.82 ± 25.5	64.17 ± 285.16	60.32 ± 282.31	<0.001

^a^ Missing data for follow up (*n* = 3). ^b^ Student’s *t*-test. ^c^ All data are expressed as pg/mL. ^d^ Peaks below the limit of quantification (LOQ) were imputed with values LOQ/2. PGE_2_: prostaglandin E_2_; 20-HETE: 20-hydroxyeicosatetraenoic acid; 12-HETE: 12-hydroxyeicosatetraenoic acid; 5-HETE: 5-hydroxyeicosatetraenoic acid.

**Table 5:** Change in circulating oxylipin concentrations by treatment group (*n* = 247).

Mean ± SD	Placebo ^a^ *n* = 123	Selenium ^a^ *n* = 130	*p*-Value ^a^
PGE_2_ ^b,c^	0.46 ± 1.6	0.32 ± 1.07	0.43
20-HETE	0.00 ± 0.04	0.00 ± 0.03	0.66
12-HETE	3.30 ± 1.2	1.70 ± 10.7	0.30
5-HETE	99.1 ± 381.9	19.3 ± 84.1	0.02

^a^ Missing data for follow-up oxylipins concentrations: placebo group (*n* = 2); selenium group (*n* = 1). ^b^ All data are expressed as pg/mL. ^c^ Peaks below the limit of quantification (LOQ) were imputed with values LOQ/2. PGE_2_: prostaglandin E_2_; 20-HETE: 20-hydroxyeicosatetraenoic acid; 12-HETE: 12-hydroxyeicosatetraenoic acid; 5-HETE: 5-hydroxyeicosatetraenoic acid.

## Data Availability

The data presented in this study are available on request from the corresponding author. The data are not publicly available due to participant privacy.
